# Identifying and resolving ethical challenges related to the enrollment of black individuals with chronic kidney disease in U.S. health registries and clinical research: a qualitative study

**DOI:** 10.3389/fepid.2026.1753620

**Published:** 2026-08-21

**Authors:** Henry Asante Antwi, Adaeze Aroh, Deidra Crews, Rodney Elliot, Nicole Huillca, C. Daniel Mullins, Mehar Sandhu, Abdou Simon Senghor

**Affiliations:** 1 Department of Nutrition and Healthcare Management, Appalachian State University, Boone, NC, United States; 2 Department of Public Health, Slippery Rock University of Pennsylvania, Slippery Rock, PA, United States; 3 Division of Nephrology, Department of Medicine, Johns Hopkins University, Baltimore, MD, United States; 4 Department of Practice, Sciences, and Health Outcomes Research, University of Maryland Baltimore School of Pharmacy, Baltimore, MD, United States; 5 Department of Public Health, University of Maryland, College Park, MD, United States

**Keywords:** blacks, chronic kidney disease, clinical research ethics, health equity, health registries, mistrust

## Abstract

**Background:**

Chronic kidney disease (CKD) disproportionately impacts Black individuals. This disparity, coupled with historical and ongoing injustices in medical research, raises significant ethical concerns that may hinder participation in health registries and clinical research, which are critical for advancing patient-centered kidney care. This study sought to identify key ethical challenges from the perspective of Black individuals with CKD to enhance their meaningful and equitable engagement.

**Methods:**

We employed a qualitative approach guided by a pragmatist bioethical framework. We conducted three virtual town hall meetings with a sample of 24 self-identified Black adults with CKD and their caregivers, recruited from the Baltimore Metropolitan Area. Data were analyzed using thematic analysis. To ensure rigor, we employed intercoder reliability checks and member-checking with participants and a Community Advisory Board.

**Results:**

Six primary ethical areas emerged: (1) lack of transparent communication, (2) distrust in the research enterprise, (3) experiences of stigmatization and discrimination, (4) cultural insensitivity, (5) lack of respect and civility, and (6) potential harm from poor-quality staff. Participants proposed and prioritized actionable solutions, including implementing mandatory transparent communication policies, enhancing community-engaged outreach, enforcing anti-discrimination legislation, providing cultural competency training for staff, and establishing rigorous quality control measures with patient input.

**Conclusion:**

Addressing these multi-faceted ethical challenges through targeted, trust-building strategies is essential to enhance the participation of Black individuals in health registries and clinical studies. This, in turn, can lead to more equitable representation in kidney research and more generalizable findings to reduce CKD disparities.

## 1 Introduction

Chronic kidney disease (CKD) is a debilitating and costly public health issue, now widely recognized as a cardinal cardiometabolic condition due to its strong interconnections with diabetes, hypertension, and cardiovascular disease (CV) (1). The global burden of CKD is substantial, and in the United States, it affects an estimated 37 million adults (2). Globally, CKD is highly prevalent and rising: an estimated 788 million adults aged 20 years and older were living with CKD in 2023, reflecting an age-standardized prevalence of approximately 14.2% and more than a doubling in absolute cases since 1990 (3). Prevalence varies substantially by region, with the highest burden reported in North Africa and the Middle East, South Asia, sub-Saharan Africa, and Latin America and the Caribbean (3). Within the United States, CKD and other chronic diseases cluster geographically in the southeastern states, a pattern that overlaps with areas of higher poverty and a greater proportion of Black residents (4). However, this burden is not borne equally. Black and African American individuals experience a disproportionate share of the CKD spectrum, with an incidence of end-stage kidney disease (ESKD) that is roughly three times higher than that of White Americans (5). This disparity is driven by a higher prevalence of key risk factors like hypertension and diabetes, alongside the influence of social determinants of health and systemic inequities in care access and quality (6, 7).

To combat this growing epidemic, a robust and inclusive research ecosystem is paramount. This ecosystem relies on two foundational pillars: health registries and clinical research. Large-scale health registries, such as the United States Renal Data System (USRDS) and the National Kidney Foundation (NKF) Patient Network, are vital national infrastructures for collecting, analyzing, and distributing population-level information (8, 9). They provide critical data on disease trends, treatment patterns, outcomes, and disparities, thereby informing public health policy, quality improvement initiatives, and observational research. The second pillar, clinical research-encompassing clinical trials, longitudinal cohort studies, and interventions-is the engine for generating new evidence. It is through clinical research that new pharmaceuticals, devices, and care models are tested for safety and efficacy, directly shaping the future standard of care (10).

The validity and generalizability of insights derived from both registries and clinical trials are fundamentally dependent on the representativeness of their participant populations (11). When certain demographic groups are systematically underrepresented, the resulting data paints an incomplete picture. Research findings may not be applicable to the missing populations, and interventions developed from such research can inadvertently perpetuate or even exacerbate existing health disparities (12). For instance, a lack of diverse participation in clinical trials for novel CKD therapies means that the safety and effectiveness of these drugs for Black patients may not be fully understood at the point of widespread clinical use (13).

Despite the clear scientific and ethical imperative for inclusion, significant barriers persist. Investigators consistently face challenges in recruiting and retaining participants from racial and ethnic minority groups, particularly in clinical trials (14). Patients from these communities encounter a multitude of obstacles, including limited access to research-intensive academic centers, logistical burdens related to transportation and time, and insufficient awareness of research opportunities (15). Compounding these logistical and structural challenges are profound ethical concerns that disproportionately discourage participation among Black communities. These concerns are rooted in a well-documented history of exploitation and mistreatment in medical research.

The Tuskegee Syphilis Study remains the most potent symbol of such ethical breaches, wherein hundreds of Black men were deliberately denied effective treatment for syphilis for decades without their informed consent (16). This historical trauma is not a relic of the past but is reinforced by contemporary experiences of discrimination, bias, and disrespect within the healthcare system (17, 18). The resulting legacy is a deep-seated and rational mistrust of the medical research enterprise (19). This mistrust is compounded by specific ethical anxieties related to data privacy, the potential for stigmatization, a lack of transparency regarding how personal health information will be used, and fears that participation may lead to unforeseen costs or harms (20, 21).

While the literature acknowledges these broad barriers, there is a critical gap in qualitative, community-engaged research that specifically investigates the ethical dimensions of research participation from the perspective of Black individuals living with CKD. Most studies focus on logistical barriers or general attitudes toward research. Fewer delve deeply into the specific ethical challenges-such as perceptions of cultural insensitivity, disrespect in patient-provider-researcher interactions, or concerns about staff competency-that act as decisive deterrents to enrolling in both registries and clinical studies.

To address this gap, this study employed a deliberative bioethics approach. Deliberative methods have been proposed as a robust way to tackle complex bioethical issues by facilitating a structured understanding of problems through dialogue, consensus-building, and the recognition of diverse viewpoints (22–24). We specifically utilized the investigative framework developed by Senghor and Racine (2022), which is grounded in pragmatist philosophy and emphasizes problem-solving through collaborative engagement with affected communities (24). This framework involves three iterative phases: (1) broadening and deepening the understanding of the morally problematic situation, (2) envisioning action scenarios, and (3) coming to a judgment based on the comparative evaluation of these scenarios.

By leveraging this ethical deliberation process with Black patients and caregivers directly affected by CKD, this research aims to move beyond simply cataloging barriers. It seeks to collaboratively identify the core ethical challenges and, most importantly, to develop and prioritize community-informed, actionable solutions. The ultimate goal is to generate a practical roadmap for researchers, registry operators, and institutional review boards to overcome these challenges, thereby fostering a more equitable, ethical, and trustworthy research environment that earns the participation of Black communities. Given the study's exploratory, single-site design, these findings are intended to generate hypotheses and illustrative, community-informed directions rather than to establish generalizable or definitive practice guidelines; we discuss this scope further in the Limitations section.

## 2 Methodology

### 2.1 Study design

This qualitative study employed a series of town hall discussions to identify and resolve ethical challenges related to the enrollment of Black individuals with CKD in U.S. health registries and clinical research. A qualitative approach was selected for its strength in capturing rich, detailed insights directly from participants on sensitive topics such as mistrust and discrimination (25, 26). The study was guided by the pragmatist bioethical deliberation framework of Senghor and Racine (24), which emphasizes problem-solving through collaborative dialogue with affected communities. The research team included individuals with expertise in bioethics, pharmacy, medicine, and public health. While the team was racially diverse, the Principal Investigator (PI) and primary facilitator for the town halls self-identifies as Black. We acknowledge that interviewer-participant concordance can influence data collection in research on race and ethnicity; having a Black facilitator was a deliberate choice to potentially foster a greater sense of safety and shared understanding among participants (27). Ethical approval was obtained from the University of Maryland Institutional Review Board (IRB HP-00107720). Informed consent was obtained from all participants, who were assured of confidentiality and the voluntary nature of their participation.

### 2.2 Participant recruitment and characteristics

Participants were recruited using snowball sampling, a method chosen due to the sensitive nature of the topic and the historical mistrust within the target population. This approach leverages existing community networks to identify participants who might otherwise be difficult to reach through traditional methods (28). Recruitment began through partnerships with local community health centers and patient advocacy groups in the Baltimore Metropolitan Area. Eligible participants were adults (≥18 years) who self-identified as Black or African American and either had a self-reported or physician-diagnosis of CKD (any stage) or were a caregiver for such an individual.

### 2.3 Data collection

Three 90-minute virtual town hall meetings were conducted via Zoom. While virtual platforms can exclude those with limited technology access (29), this format was chosen for feasibility and during the ongoing considerations for in-person gatherings. Each meeting corresponded to a phase of the deliberative framework. The first meeting focused on problem identification: participants discussed ethical challenges related to registry and clinical trial enrollment, guided by a semi-structured question guide (see Appendix A) that included questions such as “What concerns would you have about sharing your health information with a kidney disease research registry or joining a clinical trial?” and “Can you describe any past experiences, good or bad, that would influence your decision to join a research study?” The second meeting addressed scenario envisioning: the ethical issues identified in the first meeting were presented back to participants, who then proposed and discussed potential solutions. The third meeting focused on judgment: participants evaluated, refined, and selected the solutions they believed were most aligned with their values and most likely to be effective in addressing the identified challenges. The meetings were facilitated by the PI, audio-recorded, and professionally transcribed. Participants received a $50 gift card for each session they attended. This chronological structure allowed participants to move from articulating their lived experiences and concerns, to imagining ideal research environments, and finally to critically evaluating and prioritizing actionable strategies for reform.

### 2.4 Data analysis

Thematic analysis was employed to identify, analyze, and report patterns (themes) within the data (30). Two independent researchers independently coded the qualitative data using a coding framework organized around the bioethical principles of respect for persons, beneficence, and justice, first familiarizing themselves with the data through repeated reading of the transcripts, then generating initial codes using NVivo software; these codes were collated into potential themes, which were iteratively reviewed, refined, and subsequently defined and named.

Intercoder reliability was assessed with an overall Cohen's Kappa score of 0.8605, indicating substantial agreement. Reliability was also examined separately for each of the six themes; theme-specific kappa coefficients ranged from 0.790 to 1.000, indicating substantial to perfect intercoder agreement across all themes (Table 1). Discrepancies were resolved through discussion until consensus was reached. To enhance credibility, a summary of the findings was shared with participants and CAB members in a member-checking process to ensure the interpretations accurately reflected their experiences and perspectives (31).

**Table 1:** Intercoder reliability by theme.

Bioethical Principle	Ethical Challenge/Theme	Cohen's *κ*	Level of Agreement
Respect for Persons (I)	Lack of Transparent Communication	0.790	Substantial
Respect for Persons (II)	Lack of Respect and Civility	0.875	Almost perfect
Beneficence	Potential Harm from Poor-Quality Staff	0.833	Almost perfect
Respect for Persons III	Distrust	0.790	Substantial
Justice I	Stigmatization, Discrimination, and Treatment Inequality	1.000	Perfect
Justice II	Cultural Insensitivity	0.875	Almost perfect

## 3 Results

### 3.1 Profile of participants

A total of 24 participants were enrolled and assigned to one of three town hall meetings (8 participants each). We collected basic demographic and health characteristics to contextualize the sample (presented in the Results section, Table 2, in keeping with standard reporting conventions). The sample included a range of ages, genders, and CKD stages. All participants reported an annual household income of less than $80,000, with the majority (75%) earning less than $40,000. Nearly half (46%) had prior experience participating in some form of health research. A Community Advisory Board (CAB) of five members-comprising community leaders, patient advocates, an academic bioethicist, and caregivers-supervised the project. The CAB provided guidance on study design, ethical considerations, and data interpretation. Table 3 summarizes the ethical issues, proposed solutions, and preferred solutions selected by participants based on bioethical principles. These findings are discussed in detail in Section 3.2.

**Table 2:** Participant characteristics (*N* = 24).

Characteristic	n (%)
Age (years)	
18–35	4 (17%)
36–55	10 (42%)
56–75	8 (33%)
>75	2 (8%)
Gender	
Female	15 (63%)
Male	9 (37%)
Role	
Patient	18 (75%)
Caregiver	6 (25%)
Self-Reported CKD Stage	
Stage 1–2	5 (21%)
Stage 3–4	11 (46%)
Stage 5 (ESKD)	6 (25%)
Unknown	2 (8%)
Annual Household Income	
<$40,000	18 (75%)
$40,000–$79,999	6 (25%)
≥$80,000	0 (0%)
Prior Research Experience	
Yes	11 (46%)
No	13 (54%)

**Table 3:** Ethical challenges, proposed solutions, and best solutions framed by bioethical principles.

Bioethical Principle	Ethical Challenge	Proposed Solutions	Prioritized Best Solution
Respect for Persons	Lack of Transparent Communication “I feel like we're often left in the dark about what's actually happening with our information… Clear, straightforward communication shouldn't be optional—it should be required.” (PTH1) “They may ask you to sign the form, but that does not mean you really understand where your information is going or who will be able to see it.” (PTH7) “I would want them to explain everything in ordinary language, including what happens to my information after the study ends.” (PTH9)	• Improve communication using clear, simple language • Implement regulations for mandatory transparency • Establish explicit consent for data sharing	Implement mandatory transparent communication policies, ensuring patients know how their data will be used, by whom, and for what purpose in both registries and clinical trials
Respect for Persons	Lack of Respect and Civility “Being talked down to or brushed off makes me feel like I don't belong in these programs.” (PTH9) “Sometimes you ask a question and the response makes you feel as though you are wasting their time. That kind of treatment makes you not want to participate.” (PTH10) “Respect means listening to me and taking my concerns seriously, even if I do not understand all the medical terms.” (PTH8)	• Train providers on respectful communication • Establish policies for patient respect and civil treatment	Train research staff on respectful communication and establish enforceable civility policies
Beneficence	Potential Harm from Poor-Quality Staff “When staff aren't properly trained, it's not just inconvenient-it's dangerous. We need to know that the people handling our care are qualified and accountable.” (PTH11) “If the person collecting my information cannot answer basic questions, I begin to wonder whether they can be trusted with something as important as my health.” (PTH13) “The institution may have a good name, but the individual staff member is the person dealing with me. That person needs to be properly trained and supervised.” (PTH15)	• Provide continuing education for staff • Use chaperones or neutral advocates • Conduct frequent quality control inspections	Conduct frequent quality control inspections with confidential patient input to ensure accountability and continuous improvement
Respect for Persons	Distrust “There's a lot of hesitation because, frankly, we've seen our communities mistreated in the past… It's not just about saying we can trust them-it's about showing it.” (PTH3) “It is difficult to separate what happened in the past from what we experience today. People need to see that something has genuinely changed before they give their trust.” (PTH11) “Trust cannot begin when you need participants and end when the study is over. Researchers need to remain connected to the community.” (PTH15)	• Support community engagement programs • Ensure transparent data use policies • Provide legal recourse for data breaches	Support sustained community engagement programs and trust-building policies that involve the community in research governance and design
Justice	Stigmatization, Discrimination, and Treatment Inequality “Sometimes, it feels like we're treated differently just because of who we are… Until everyone receives the same level of care, why would we trust these systems?” (PTH5) “As a Black patient, you sometimes feel that assumptions have already been made about you before you have had the opportunity to explain yourself.” (PTH16) “If I believe that I will receive different treatment because of my race or my income, I am not going to feel safe joining the study.” (PTH13)	• Promote inclusive policies • Develop and enforce anti-discrimination legislation	Enforce strict anti-discrimination legislation and monitoring systems to ensure fair treatment and protection for all patients
Justice	Cultural Insensitivity “It's hard to trust someone with your health information when they don't seem to understand or respect where you're coming from.” (PTH7) “You cannot come into the community only when you need people for a study. You have to understand the community and build relationships first.” (PTH17) “Cultural understanding is more than knowing that I am Black. It means understanding my concerns, my family, and why I may be cautious about research.” (PTH18)	• Provide cultural competency training for providers • Enhance CKD education in Black communities	Implement mandatory cultural competency training for research staff combined with tailored CKD education for Black communities

### 3.2 Summary of themes

#### 3.2.1 Narrative synthesis of results

##### 3.2.1.1 Meeting 1: problem identification

The first meeting was dedicated to understanding participants' baseline perceptions, concerns, and experiential knowledge regarding health registries and clinical research. The discussions revealed a complex landscape of apprehension rooted in historical mistrust, personal experiences with the healthcare system, and immediate fears about data security and differential treatment.

When asked what the terms “health registry” or “clinical trial” meant to them, participants often associated these concepts with data collection and experimentation, but many expressed uncertainty about the specific purposes and mechanisms involved. Their first thoughts upon being invited to join a registry or study were frequently characterized by hesitation and suspicion. Several participants noted that their immediate reaction would be to question the motives behind the invitation, wondering whether they were being recruited for the benefit of researchers or for the genuine improvement of their own health outcomes. This skepticism was not abstract but was grounded in a deep awareness of historical abuses and ongoing disparities in healthcare.

Concerns about sharing personal health information were paramount. Participants articulated significant anxiety about data privacy, including who would have access to their information, how it might be shared, and whether it could be used against them or their families. One participant captured this concern directly, stating, “I feel like we're often left in the dark about what's actually happening with our information” (Participant PTH1). Another participant elaborated on the inadequacy of current consent processes, observing, “They may ask you to sign the form, but that does not mean you really understand where your information is going or who will be able to see it” (Participant PTH7). The fear of data being sold to third parties or used for purposes beyond the stated research aims was a recurring theme. Additionally, many participants expressed concern that their information might be used to discriminate against them in other contexts, such as employment or insurance.

Past experiences with the healthcare system emerged as powerful determinants of their willingness to participate. Participants recounted a spectrum of experiences, from feeling dismissed or disrespected by providers to witnessing or hearing about community members being mistreated in research settings. These experiences were not merely historical anecdotes but were lived realities that shaped their current perspectives. One participant poignantly reflected on the difficulty of separating past injustices from present-day interactions, stating, “It is difficult to separate what happened in the past from what we experience today. People need to see that something has genuinely changed before they give their trust” (Participant PTH11). Another participant emphasized that trust cannot be assumed, noting, “There's a lot of hesitation because, frankly, we've seen our communities mistreated in the past. It's not just about saying we can trust them, it's about showing it” (Participant PTH3).

Fears and worries extended beyond individual concerns to encompass family and community implications. Participants worried about the potential stigma associated with participating in research, particularly if it reinforced negative stereotypes about Black patients or kidney disease. They also expressed concerns about being treated differently, receiving substandard care or being subjected to assumptions based on their race or income. One participant articulated this fear eloquently, stating, “Sometimes, it feels like we're treated differently just because of who we are. Until everyone receives the same level of care, why would we trust these systems?” (Participant PTH5). Another participant noted, “As a Black patient, you sometimes feel that assumptions have already been made about you before you have had the opportunity to explain yourself” (Participant PTH16). A third participant connected this directly to research participation, observing, “If I believe that I will receive different treatment because of my race or my income, I am not going to feel safe joining the study” (Participant PTH13). These fears were compounded by a sense that cultural insensitivity pervades the research enterprise. As one participant explained, “It's hard to trust someone with your health information when they don't seem to understand or respect where you're coming from” (Participant PTH7). Another participant criticized the transactional nature of many research relationships, stating, “You cannot come into the community only when you need people for a study. You have to understand the community and build relationships first” (Participant PTH17). A third participant elaborated on the depth of cultural understanding required, noting, “Cultural understanding is more than knowing that I am Black. It means understanding my concerns, my family, and why I may be cautious about research” (Participant PTH18).

When asked what would make them feel more comfortable or safe about joining a registry or clinical trial, participants emphasized the need for clear, transparent communication; genuine respect and civility; and sustained community engagement. They articulated a desire for researchers to explain everything in ordinary language. One participant expressed this clearly, stating, “I would want them to explain everything in ordinary language, including what happens to my information after the study ends” (Participant PTH9). Another participant emphasized the importance of being heard and taken seriously, observing, “Respect means listening to me and taking my concerns seriously, even if I do not understand all the medical terms” (Participant PTH8). Trust, they emphasized, must be built over time through consistent, respectful, and transparent actions. One participant captured this sentiment powerfully, stating, “Trust cannot begin when you need participants and end when the study is over. Researchers need to remain connected to the community” (Participant PTH15).

##### 3.2.1.2 Meeting 2: scenario envisioning

Building on the concerns articulated in the first meeting, the second session invited participants to envision solutions. Researchers presented key themes from the previous discussion, including distrust, lack of transparency, discrimination, and cultural insensitivity, and asked participants to consider what researchers, hospitals, and institutions could do to address these challenges.

Participants were asked to imagine they were in charge of designing a registry or clinical trial and to propose rules that would protect people. Their responses were wide-ranging and thoughtful, reflecting a deep engagement with the ethical complexities of research participation. Proposed solutions centered on several key areas: communication, respect, staff quality, anti-discrimination measures, cultural competency, and community engagement.

In the realm of communication, participants advocated for mandatory transparency policies that would require researchers to provide clear, accessible information about data use, sharing, and storage. They emphasized that consent should be an ongoing, explicit process rather than a one-time signature, and that patients should have the right to withdraw their data at any time without penalty. One participant expressed this need for clarity succinctly, stating, “Clear, straightforward communication shouldn't be optional, it should be required” (Participant PTH1).

Regarding respect, participants proposed formal training for all research staff on respectful communication and the establishment of enforceable civility policies. They stressed that respect is not optional but is a fundamental ethical requirement, and that staff who fail to treat patients with dignity should be held accountable. One participant articulated the impact of disrespectful treatment, stating, “Being talked down to or brushed off makes me feel like I don't belong in these programs” (Participant PTH9). Another participant shared a personal experience, observing, “Sometimes you ask a question and the response makes you feel as though you are wasting their time. That kind of treatment makes you not want to participate” (Participant PTH10).

To address potential harm from poor-quality staff, participants suggested continuing education, the use of chaperones or neutral advocates during interactions, and frequent quality control inspections. They emphasized that the competence and accountability of front-line staff are critical to patient safety and trust, and that oversight mechanisms must be robust and independent. One participant highlighted the danger of inadequate training, stating, “When staff aren't properly trained, it's not just inconvenient, it's dangerous. We need to know that the people handling our care are qualified and accountable” (Participant PTH11). Another participant expressed how staff competence directly impacts trust, noting, “If the person collecting my information cannot answer basic questions, I begin to wonder whether they can be trusted with something as important as my health” (Participant PTH13). A third participant made an important distinction between institutional reputation and individual interactions, observing, “The institution may have a good name, but the individual staff member is the person dealing with me. That person needs to be properly trained and supervised” (Participant PTH15).

On the issue of discrimination and stigmatization, participants called for inclusive policies and the development and enforcement of anti-discrimination legislation. They argued that systemic change requires legal and structural backing, and that monitoring systems are essential to ensure that all patients receive fair and equitable treatment.

Cultural insensitivity was addressed through proposals for mandatory cultural competency training for all research staff, combined with tailored CKD education for Black communities. Participants insisted that researchers must understand the communities they serve, not superficially, but with genuine awareness of their concerns, values, and historical experiences. The quotes from the first meeting around cultural understanding were revisited and reinforced as foundational to any meaningful solution.

Finally, participants emphasized the importance of community engagement, proposing sustained programs that involve the community in research governance and design. They argued that trust cannot be episodic but must be nurtured through ongoing relationships, shared decision-making, and meaningful inclusion of community voices at every stage of the research process.

##### 3.2.1.3 Meeting 3: judgment and prioritization

In the final meeting, participants were asked to evaluate the proposed solutions, identify those they deemed most effective, and prioritize a select few for immediate implementation. This judgment phase was critical for translating the wealth of ideas from the second meeting into actionable, feasible strategies.

When asked which solutions would be most effective, participants consistently prioritized those that addressed the root causes of distrust and power imbalance. They emphasized that transparency, accountability, and sustained community engagement were non-negotiable foundations for any ethical research enterprise. Communication policies that mandate clear, ongoing, and accessible information sharing were seen as essential for restoring patient agency and informed decision-making.

Participants also evaluated the practicality of the proposed solutions. While many acknowledged the value of training and education, some questioned whether these measures would be sufficient without robust enforcement mechanisms. They expressed skepticism that voluntary training programs or aspirational policies would lead to meaningful change, arguing instead for mandatory requirements with clear consequences for non-compliance. Similarly, while cultural competency training was widely supported, participants stressed that it must be accompanied by structural changes, such as diverse hiring practices and community representation on research oversight boards.

When asked to identify the highest priorities, participants converged on three interconnected solutions that they believed would have the greatest impact. First, they prioritized the implementation of mandatory transparent communication policies, ensuring that patients know exactly how their data will be used, by whom, and for what purpose in both registries and clinical trials. This was seen as the bedrock of informed consent and trust, directly responding to participants' earlier concerns about being “left in the dark.”

Second, they prioritized sustained community engagement programs and trust-building policies that involve the community in research governance and design. Participants argued that researchers must remain connected to the community before, during, and after studies, and that community voices must be integrated into decision-making processes to ensure that research is responsive to community needs and values. This priority directly addressed the concern that researchers “cannot come into the community only when you need people for a study” (Participant PTH17).

Third, they prioritized the enforcement of strict anti-discrimination legislation and monitoring systems to ensure fair treatment and protection for all patients. This was viewed as a structural safeguard against the stigmatization and inequality that have historically characterized healthcare and research for African American communities, directly responding to participants' fears about being treated differently “just because of who we are” (Participant PTH5).

Additional priorities included frequent quality control inspections with confidential patient input to ensure staff accountability, and mandatory cultural competency training for research staff combined with tailored CKD education for Black communities. Participants viewed these as complementary measures that would reinforce the broader goals of transparency, respect, and equity. The emphasis on staff training and accountability directly addressed participants' concerns that “the individual staff member is the person dealing with me” (Participant PTH15) and that “that person needs to be properly trained and supervised.”

Ultimately, the most important message participants wished to convey to registry and clinical trial creators was that trust is not a given but must be earned through consistent, transparent, and respectful action. They called for researchers to recognize the historical and systemic contexts that shape community skepticism, to engage meaningfully with patients as partners rather than subjects, and to demonstrate a genuine commitment to equity and justice that extends beyond the lifespan of any single study. As one participant succinctly stated, “Trust cannot begin when you need participants and end when the study is over. Researchers need to remain connected to the community” (Participant PTH15). This sentiment encapsulates the overarching theme of the discussions: that ethical research requires not only procedural safeguards but also a fundamental transformation in how researchers relate to and respect the communities they seek to engage.

## 4 Discussion

This study provides a detailed examination of the ethical challenges Black individuals with CKD perceive in the context of enrolling in health registries and clinical research. Our findings, grounded in participant voices, highlight that the barriers are not merely logistical but are fundamentally rooted in issues of trust, respect, and justice. By organizing our results around the core bioethical principles of respect for persons, beneficence, and justice (32), we can better align these community-identified concerns with established ethical frameworks and propose more targeted interventions. The challenges of transparent communication and respect and civility fall squarely under the principle of respect for persons, which mandates recognizing the autonomy and dignity of individuals. Participants felt this respect was absent when communication was opaque or interactions were dismissive. This aligns with existing literature where poor communication exacerbates mistrust and disengagement (33). The proposed solution-mandatory transparency policies and civility training-are not simply about mitigating mistrust but are foundational to gaining trust by demonstrating respect for participants as partners and rightful owners of their health data.

The principle of beneficence, or the obligation to maximize benefits and minimize harm, is directly threatened by the potential for harm from poor-quality staff. Participants' concerns about safety and competence underscore that enrollment in a registry or trial is not a passive act; it involves interactions with a system that must be competent and safe. The prioritized solution of quality control inspections with confidential patient feedback creates a system of accountability and continuous improvement, actively demonstrating a commitment to patient welfare and thereby building trust. Finally, the challenges of, discrimination, and cultural insensitivity are issues of justice, which requires the fair distribution of benefits and burdens of research. The profound distrust described by participants is a rational response to historical and ongoing injustices (16, 18). Therefore, solutions must be systemic and restorative. Community engagement programs and anti-discrimination legislation are not merely protective; they are proactive mechanisms to redress power imbalances and ensure equity. Similarly, cultural competency training is a step toward ensuring that the research apparatus is as responsive to Black communities as it is to others. These actions move beyond mitigating mistrust and begin the work of earning trust through demonstrated fairness and shared power.

These findings resonate with, and extend, a growing body of work on research participation among Black and other historically underrepresented communities. Consistent with prior systematic reviews, our participants described logistical, informational, and relational barriers to enrollment that mirror those catalogued across African American, Latino, Asian American, and Pacific Islander populations (34), while our emphasis on transparency and civility echoes long-standing accounts of how the legacy of Tuskegee and related abuses continues to shape contemporary mistrust of research institutions (35). Two recent qualitative studies further reinforce our findings: work examining contextual factors in genomic screening and research participation among Black and White Americans similarly identified trust, communication, and perceived fairness as decisive factors in enrollment decisions (36), and related work on the trustworthiness of medical information and genetic testing found that perceived bias in how information is delivered-not only its content-shapes Black patients' willingness to participate (37). Together, this literature and our findings suggest that trust-building interventions must address both the structural fairness of research systems and the interpersonal quality of researcher-participant interactions.

## 5 Conclusion

This study identifies a core set of ethical challenges that act as significant barriers to the enrollment of Black individuals in CKD registries and clinical research. The findings make it clear that overcoming these barriers requires more than just simplified forms or better marketing; it demands a fundamental shift toward practices that actively build trust through demonstrated respect, competence, and justice. The solutions prioritized by participants-transparent communication, community engagement, anti-discrimination measures, cultural competency, respect, and quality assurance-provide a concrete roadmap for researchers, registry operators, and policymakers. By adopting these community-informed, trust-building strategies, the research community can take meaningful steps toward achieving equitable representation in kidney disease research, ultimately leading to more effective and inclusive care for all.

In practice, these findings suggest concrete starting points for action: research registries and institutional review boards (IRBs) can adopt standardized, mandatory data-transparency disclosures at the point of consent; academic health systems can tie staff credentialing and quality assurance to confidential patient feedback; and funders and policymakers can condition support for CKD research on demonstrated community-engagement infrastructure. We offer these as illustrative starting points for local adaptation rather than a one-size-fits-all protocol, consistent with the exploratory scope of this study.

### 5.1 Limitations and future research

This study has several limitations. First, the use of snowball sampling and a virtual format, while necessary for recruitment, may limit the diversity of perspectives. Our sample was from a single geographic area and, despite having a range of incomes, was predominantly low-income, making it difficult to fully disentangle the effects of ethnicity from socioeconomic status. The cross-sectional design provides only a snapshot in time. Furthermore, while having a Black facilitator was a strength, we did not systematically collect data on how interviewer-participant concordance specifically influenced responses. Because the sample comprised fewer than 30 participants drawn from a single metropolitan area, the specific recommendations prioritized by our participants should be understood as exploratory and hypothesis-generating rather than as a validated, generalizable roadmap; broader or policy-level application would require confirmation in larger and more geographically diverse samples.

Future research should include larger, more geographically and socioeconomically diverse samples to explore the intersection of race, class, and other factors. Quantitative studies are needed to validate the prevalence of these ethical challenges. Most importantly, implementation science is needed to pilot and evaluate the proposed trust-building interventions, such as community-led research advisory boards or standardized transparency protocols, to assess their real-world effectiveness in improving enrollment equity and trust across both registries and clinical trials.

## Data Availability

The raw data supporting the conclusions of this article will be made available by the authors, without undue reservation.

## Appendix A: Semi-Structured Question Guide for Town Hall Meetings

Meeting 1: Problem Identification

• What does the term “health registry” or “clinical trial” mean to you?

• What would be your first thoughts if your doctor asked you to join a registry or a research study for kidney disease?

• What concerns, if any, would you have about sharing your personal health information with a registry or research team?

• Can you describe any past experiences—positive or negative—with the healthcare system or research that would influence your decision to join?

• What fears or worries might come up for you or your family when considering joining a study?

• What would make you feel more comfortable or safe about joining a registry or clinical trial?

Meeting 2: Scenario Envisioning

• Last time, we talked about challenges like [list key themes from Meeting 1]. Thinking about [specific challenge, e.g., distrust], what could researchers or hospitals do to address this?

• If you were in charge of designing a registry or clinical trial, what rules would you put in place to protect people?

• Imagine a research study that you would feel proud and safe to be a part of. What would it look like? How would it operate?

Meeting 3: Judgment

• We've discussed several possible solutions, such as [list proposed solutions]. Which of these do you think would be most effective?

• Are there any of these solutions that seem impractical or unlikely to work? Why?

• If we could only implement a few of these ideas, which ones should be the highest priority and why?

• What is the most important thing registry and clinical trial creators need to understand about your community's perspective?
